# Complete Genome Sequence of *Gordonia* phage RedRaider

**DOI:** 10.17912/micropub.biology.001874

**Published:** 2026-01-05

**Authors:** Peyton Abraham, Princess Bola-Lawal, Mary Britten, Randi Childress, Abby Flannagan, Mallory Gramstad, Hayden Groos, Sharon Huffman, Korver Hupke, Corbin Kleis, Jenna Kluxdal, Ayda Maassen, Caden Meyer, Elsa Meyer, Logan Miller, Emma Steinhardt, Travis Sweeney, Alexa Trover, Shayler Van Gelder, Dane Schoenborn, Byron Noordewier, Sara Tolsma

**Affiliations:** 1 Biology, Northwestern College - Iowa, Orange City, Iowa, United States

## Abstract

We isolated and annotated a novel bacteriophage, RedRaider, on *Gordonia terrae* 3612. Annotation revealed genome features typical of cluster CR3 phages with the putative lysin A function encoded in two gene products, a protease C39 domain and a glycosyl hydrolase domain, and lysin B encoded distally in the left arm of the genome.

**Figure 1. Phage RedRaider:**
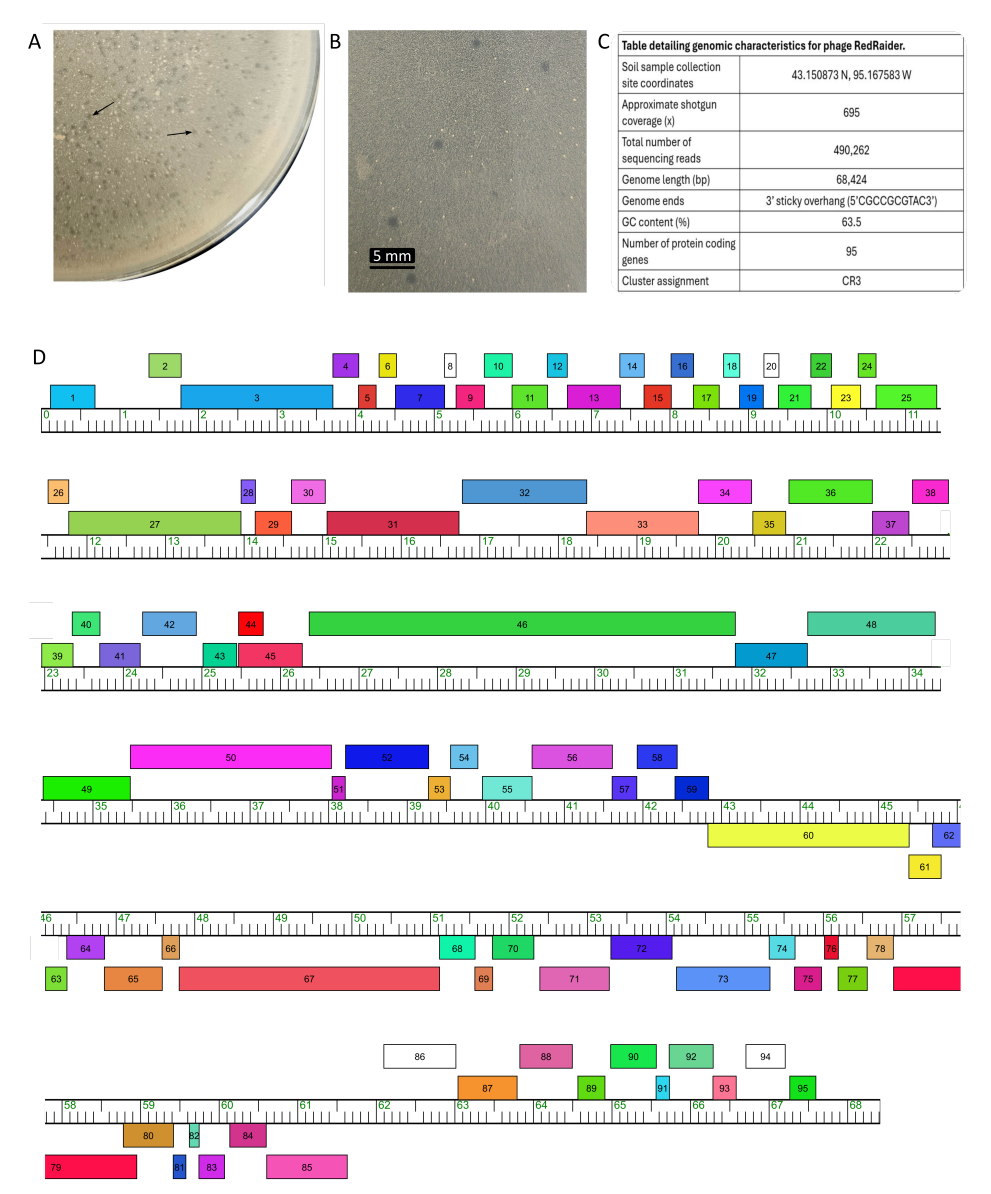
A. Picture of 1/4 of a 100 mm whole plate plaque assay of phage RedRaider. Arrows identify representative plaques (dark) to distinguish plaques from bacterial clumps in the lawn which appear white. B. RedRaider plaque assay at lower dilution for detailed plaque morphology. Plaques were clear with turbid, irregular edges and ranged from 0.8 to 2.3 mm in diameter (n = 10) with a mean diameter of 1.2 mm. Scale bar = 5 mm. C. Genomic characteristics of phage RedRaider. D. Phamerator map of RedRaider.&nbsp; Colored boxes indicate annotated genes.&nbsp; The ruler indicates the position in the genome.&nbsp; Boxes above the ruler represent genes in the forward direction; boxes below the line represent genes in the reverse direction.

## Description

Bacteriophages are a rapidly evolving group of infectious particles (Hendrix 2002). They are remarkably abundant, found in nearly every ecosystem investigated (Rohwer et al., 2014). In this paper, we report the genomic organization of RedRaider, a newly discovered member of this dynamic population.

We isolated RedRaider from 15 g of very dry, surface topsoil that was near a patch of grass clippings using standard methods (see Figure 1C for GPS coordinates) (Poxleitner et al, 2018). Briefly, the soil sample was washed with peptone-yeast extract-calcium (PYCa) liquid medium supplemented with 0.1% dextrose and the suspension was filtered (0.2-µm pore size). The filtrate was inoculated with *Gordonia terrae* 3512 and incubated, with shaking, at 30 ^o^ C for two days. The resulting culture was filtered (0.2- µm pore size) to remove bacteria and phages in the filtrate were plated in PYCa top agar with *G. terrae.* Plaques of RedRaider were observed after 24 hours and purified with three rounds of plating. RedRaider formed small plaques, 0.8 - 2.3 mm in diameter (n = 10), with slightly turbid, irregular edges after 24-48 hours at 30 ^o^ C ( Figure 1A, B).

We isolated phage DNA from high titer lysate using Wizard DNA Clean-Up System (Promega) then concentrated the DNA with a DNA Clean and Concentrator Kit (ZYMO Research). RedRaider DNA was pooled with DNA isolated from a mycobacteriophage and a microbacterium phage and then prepared for sequencing on an Illumina NextSeq 1000 sequencer using the NEB Ultra II Library kit. The resulting 100 base raw reads were trimmed with cutadapt 4.7 (using the option: –nextseq-trim 30) and filtered with skewer 0.2.2 (using the options: -q 20 -Q 30 -n -l 50) prior to assembly (Martin 2011; Jiang et al, 2014; Wick et al, 2017; Gordon et al, 1998). Genome ends were determined with Consed (v29.0) using default settings (Gordon and Green 2013; Russell 2018). The DNA sequences from each pooled sample were substantially different, which enabled the assembly of three distinct genomes, one of which was RedRaider (Russell 2018). RedRaider was assigned to cluster CR3 based on gene-content similarity (GCS) of 35% or higher to sequenced bacteriophages present in the Actinobacteriophage database, phagesDB (Russell and Hatfull 2017) using the phagesDB GCS tool and previously described criteria (Hatfull et al., 2010). Sequencing details and genome characteristics are reported in Figure 1C .

RedRaider's genome was autoannotated using DNA Master v5.23.2 (cobamide2.bio.pitt.edu) with embedded Glimmer v3.02 (Delcher et al., 2007) and Genemark v2.5 (Besemer and Borodovsky 2005). The autoannotation was manually refined using these tools along with several others. Start site conservation was analyzed using Starterator (hhtp://phages.wustl.edu/starterator/). BLAST, using the Actinobacteriophage and NCBI non-redundant database (Altschul et al., 1990), Phamerator using Actino draft database v578 (Cresawn et al., 2011), and HHPRED, using the PDB_mmCIF70, Pfam- v.36, NCBI Conserved Domains databases (Söding et al., 2005) were used to make putative functional calls. We used Aragorn v1.1 (Laslett et al., 2004) and tRNAscan-SE v2.0 (Lowe et al., 1997) to scan for tRNA genes and deepTMHMM to identify putative transmembrane proteins (Hallgren et al., 2022). Many of the programs we used embedded in PECAAN (discover.kbrinsgd.org) and we used default parameters for all software programs. Our annotation identified 95 putative protein-coding genes and no tRNA genes ( Figure 1C, D).

RedRaider's genome organization is typical of bacteriophages with structural genes located in the left arm of the genome while genes encoding transcription factors and nucleic acid modification enzymes are primarily located in the right arm of the genome (Hatfull and Hendrix 2011). Genes are transcribed in both directions in the genome with 69 genes transcribed in one direction and the remaining 26 transcribed in the opposite direction ( Figure 1D ). We annotated four orphams, which are genes for which no homologues exist within the Actinobacteriophage database: genes 8, 20, 86, and 94.

Like the other CR3 cluster phages, RedRaider has a non-canonical lysis cassette organization (Pimentel 2014). The putative lysin B gene (gp25) is located in the left arm of the genome, upstream of genes encoding putative structural proteins such as major capsid, tape measure, and minor tail proteins. The putative lysin A gene (gp 55 and gp 56) is located downstream of the putative structural genes, as expected, and is encoded by two gene products, one encoding the protease C39 domain and another the glycosyl hydrolase domain ( Figure 1D ). We were unable to identify a gene encoding holin function but noted three putative gene products (gp57, gp58, and gp59) with predicted transmembrane domains encoded immediately downstream of the two lysin A genes (Bavda et al., 2021).

RedRaider represents only the sixth cluster CR3 phage from the Actinobacteriophage database discovered making this work an important contribution to our growing understanding of phage diversity, genomic structure, and evolution (Dion et al., 2020).

**Data Availability.**

RedRaider is available at GenBank with Accession No. PV329801 and Sequence Read Archive (SRA) No. SRX28484027 .

**Table:** 
